# The Contribution of the Skin Microbiome to Psoriasis Pathogenesis and Its Implications for Therapeutic Strategies

**DOI:** 10.3390/medicina60101619

**Published:** 2024-10-03

**Authors:** Diana Sabina Radaschin, Alin Tatu, Alina Viorica Iancu, Cristina Beiu, Liliana Gabriela Popa

**Affiliations:** 1 Department of Clinical Medical, Faculty of Medicine and Pharmacy, “Saint Parascheva” Infectious Disease Clinical Hospital, Multidisciplinary Integrated Centre of Dermatological Interface Research Centre (MICDIR), “Dunarea de Jos” University of Galati, 800008 Galati, Romania; 2 Department of Morphological and Functional Sciences, “Dunarea de Jos” University of Galati, 800008 Galati, Romania; 3 Department of Oncologic Dermatology, Elias Emergency University Hospital, Carol Davila University of Medicine and Pharmacy, 020021 Bucharest, Romania

**Keywords:** skin microbiome, psoriasis, chronic inflammatory skin disease, probiotics, prebiotics

## Abstract

Psoriasis is a common chronic inflammatory skin disease, associated with significant morbidity and a considerable negative impact on the patients’ quality of life. The complex pathogenesis of psoriasis is still incompletely understood. Genetic predisposition, environmental factors like smoking, alcohol consumption, psychological stress, consumption of certain drugs, and mechanical trauma, as well as specific immune dysfunctions, contribute to the onset of the disease. Mounting evidence indicate that skin dysbiosis plays a significant role in the development and exacerbation of psoriasis through loss of immune tolerance to commensal skin flora, an altered balance between Tregs and effector cells, and an excessive Th1 and Th17 polarization. While the implications of skin dysbiosis in psoriasis pathogenesis are only starting to be revealed, the progress in the characterization of the skin microbiome changes in psoriasis patients has opened a whole new avenue of research focusing on the modulation of the skin microbiome as an adjuvant treatment for psoriasis and as part of a long-term plan to prevent disease flares. The skin microbiome may also represent a valuable predictive marker of treatment response and may aid in the selection of the optimal personalized treatment. We present the current knowledge on the skin microbiome changes in psoriasis and the results of the studies that investigated the efficacy of the different skin microbiome modulation strategies in the management of psoriasis, and discuss the complex interaction between the host and skin commensal flora.

## 1. Introduction

Psoriasis is a common chronic inflammatory skin disease, with an estimated worldwide prevalence of 2–3% [1]. The geographic distribution of psoriasis varies greatly. While it is seldom encountered in countries near the Equator, in Northern European countries, its prevalence is as high as 8–11% [2]. Psoriasis equally affects both genders [3]. It can occur at any age, but it is very uncommon in children, with a prevalence of 0–1.4% in the pediatric population [4]. In most cases, psoriasis onset takes place either in the 30–39 years or in the 50–69 years age groups [3]. It is more frequent in Caucasians compared to Afro-Americans and Asians [3,5].

Psoriasis represents much more than a simple inaesthetic skin disease, the spectrum of clinical manifestations of psoriasis comprising not only cutaneous lesions, but also mucosal lesions, nail alterations, psoriatic arthritis, and a series of frequently associated comorbidities, such as obesity, diabetes, dyslipidemia, arterial hypertension, metabolic syndrome, cardiovascular diseases, inflammatory bowel disease, nonalcoholic fatty liver disease, anxiety, and depression [6,7,8]. These associations are explained by common genetic susceptibility, common risk factors, or shared immune and inflammatory pathogenic pathways [8].

The clinical hallmark of psoriasis is the typical well-demarcated, erythematous plaque covered by fine, silvery scales especially distributed on the extensor aspects of the limbs, the scalp, umbilical, and lumbosacral areas [9]. Nevertheless, apart from chronic plaque psoriasis, the disease can present in many clinical forms, including guttate psoriasis, characterized by eruptive small, usually infracentrimetric skin lesions, inverse psoriasis, which affects flexural areas, and pustular psoriasis, in which the erythematous plaques are covered by initially sterile pustules, erythrodermic psoriasis, or nail psoriasis [9]. Psoriatic arthritis accompanies the skin lesions in 10–30% of patients [9].

Genetic predisposition, environmental factors like smoking, alcohol consumption, psychological stress, consumption of certain drugs, and mechanical trauma, as well as specific immune dysfunctions, contribute to the onset of the disease [9]. Psoriasis may be triggered by environmental exposures, especially infectious agents or by unmasked autoantigens, such as keratins 17 and 13 or neuropeptides like substance P, heterogenous nuclear ribonucleoproteins, cathelicidin, LL-37, A disintegrin and metalloprotease domain containing thrombospondin type 1 motif-like 5 (ADAMTSL5), phospholipase A2 group IVD, and pso p27 [10,11]. Damage-associated molecular patterns are recognized by pattern recognition receptors (PRRs) expressed by dendritic cells, inducing their activation and maturation and the subsequent Th1 and Th17 polarization of the immune response. Attracted to the skin, Th1 and Th17 cells release large amounts of tumor necrosis factor (TNF) α, interleukin (IL) 1, IL-2, interferon (IFN) γ and IL-17 A/F, IL-21, and IL-22, respectively, leading to massive local and systemic inflammation, and keratinocyte proliferation [12]. Keratinocytes and cells of the innate immune system, like γδ T cells, natural killer (NK) cells, NK-T cells, innate lymphoid cells, macrophages, and neutrophils, also actively contribute to the inflammatory state by the release of cytokines and chemokines [12]. Thus, psoriasis vulgaris is a model of Th1/Th17-mediated immune disease.

The pathogenesis of pustular psoriasis, on the other hand, is principally mediated by autoinflammatory and innate immune responses [13]. The dominant effector cytokine in this particular form of psoriasis is IL-36, a member of the IL-1 family [13]. IL-36 exerts autocrine effects on keratinocytes, increasing IL-36 production and the secretion of other proinflammatory cytokines, antimicrobial peptides (AMPs), and neutrophil chemoattractants [14]. IL-36 also activates DCs and promotes their maturation and the production of proinflammatory cytokines like IL-1, IL-6, TNF-α, and IL-23 [15,16]. IL-36 acts on CD4+ T cells, inducing their proliferation and stimulating the release of IFN-γ, IL-4, and IL-17 [15,17].

Although tremendous progress has been made in the understanding of psoriasis pathogenesis during the last decades, there are still numerous unsolved issues, and psoriasis continues to represent a hot topic of research. Novel biologic and small-molecule treatments are remarkably effective in psoriasis. Nevertheless, the course of the disease is highly unpredictable, and the majority of patients experience recurrences even after long periods of complete clinical remission. As no curative treatment currently exists for this very common disease, it continues to represent a public health issue, being associated with significant morbidity, a major impact on the patients’ quality of life, and considerable economic costs [18]. Therefore, the identification and correction of risk factors for psoriasis, the uncovering of new therapeutic targets, and implementation of innovative treatment strategies still represent major research objectives. Recent studies shed light on the pivotal role of the skin microbiome in the maintenance of skin homeostasis and the implication of cutaneous dysbiosis in the development of numerous dermatoses, including psoriasis [19]. Although it is easily apprehensible that loss of immune tolerance to skin commensal flora leads to inflammation and aggravates oxidative stress [20,21,22,23,24,25], both favoring psoriatic disease, the influence of dysbiosis on psoriasis pathogenic mechanisms is far more complex and is just starting to be unveiled. This review offers a synthesis of the current state of knowledge on this topic, discusses the research limitations and gaps in this field, and presents the future perspectives in the prevention and adjuvant treatment of psoriasis by skin microbiome modulation.

## 2. The Composition and Function of the Skin Microbiome

**Figure 1:**
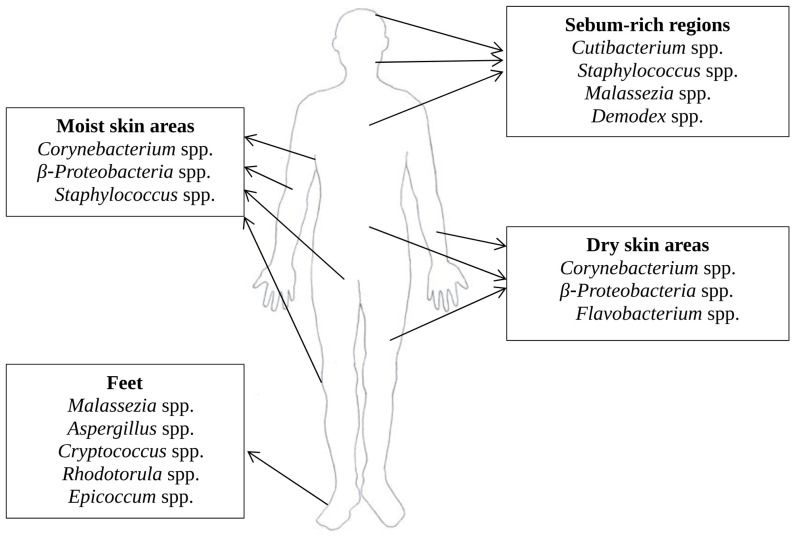
Distribution of the skin commensal flora depending on local conditions.

Compared to the colon, which, given its richness in nutrients, harbors the highest density and diversity of microorganisms, being colonized by approximately 1014 bacterial cells [26] comprising circa 3000 species [27,28], the skin’s commensal flora is less abundant, but remarkably diverse, counting over 100 phylotypes [29]. It comprises not only skin-resident aerobic and anaerobic Gram-positive bacteria (mainly *Corynebacterium* spp., *Cutibacterium* spp., *Streptococcus* spp., *Staphylococcus* spp., *Actinobacteria* spp., and *Firmicutes* spp.) and Gram-negative facultative or obligate anaerobic bacteria (primarily *Proteobacteria* spp. and *Bacteroidetes* spp.), but also bacterial species characteristic to the gut microbiome, such as *Escherichia* spp., *Enterobacter* spp., and *Enterococcus* spp. [29,30,31,32]. The distribution of the commensal flora varies depending on the local conditions, being greatly influenced by the skin’s humidity, temperature, lipid content, and light exposure (Figure 1) [24]. While Corynebacterium spp. and *β-Proteobacteria* spp. colonize both moist and dry skin areas, *Staphylococcus* spp. preferentially colonizes moist and sebum-rich regions, *Flavobacterium* spp. is predominately isolated from dry regions and *Cutibacterium* spp. is present in higher numbers in areas rich in sebaceous glands [33,34]. Fungi are also an important part of the skin microbiome. *Malassezia* spp. abounds in sebum-rich areas [35], whereas the skin of the feet is populated by a variety of fungi, such as *Malassezia* spp., *Aspergillus* spp., *Cryptococcus* spp., *Rhodotorula* spp., and *Epicoccum* spp., given the favorable local conditions [35]. Pilosebaceous units are also colonized by *Demodex* spp., a commensal arthropod generally detected on the face and scalp [36,37,38]. Viruses such as human papilloma virus (HPV), particularly Papillomaviridae β, Polyomaviridae, and Circoviridae, are a less prominent part of the skin microbiome [33,39].

The human microbiome begins to build up in utero [40] and progressively diversifies and matures after birth into organ-specific microbiomes [41], playing a pivotal role in the infant’s normal growth and development [42]. The composition of the skin microbiome substantially changes during infancy due to gradual exposure to environmental factors [43,44,45] and during puberty as a result of the marked androgen-dependent increase in the activity of sebaceous glands, which promotes the growth of lipophilic bacteria and *Malassezia* spp. [33,34,35,46]. It tends to remain stable during adulthood [33] in the absence of major external or internal influences, such as altered nutrition, deficient or excessive hygiene, antimicrobial topical or systemic treatments, significant climate changes, and comorbidities [34,47,48,49,50].

**Figure 2:**
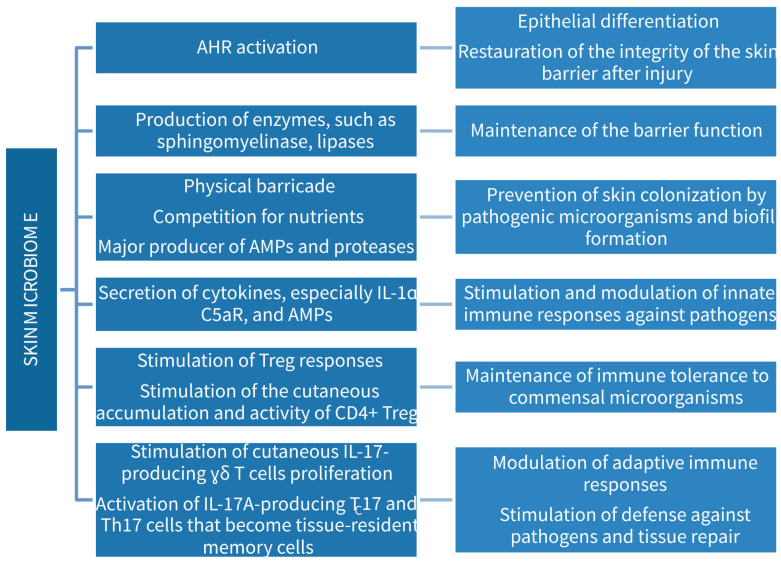
The role of skin microbiome in skin homeostasis.

A balanced skin microbiome is essential for maintaining an efficient skin barrier, as it intervenes in a plethora of physical, chemical, and immunological processes (Figure 2) [51,52]. Metabolites of the commensal bacteria activate the aryl hydrocarbon receptor (AHR) expressed by keratinocytes, which is essential for epithelial differentiation and restauration of the integrity of the skin barrier after injury [53]. Commensal bacteria also secrete enzymes with important roles in the maintenance of the barrier function. *Staphylococcus epidermidis* secretes sphingomyelinase, which generates ceramides, an important part of the stratum corneum lipid bilayer and at the same time releases nutrients that sustain the microbiome [54]. Lipophilic bacteria like *Cutibacterium acnes* and *Corynebacterium* spp. produce lipases that act on triglycerides present in the sebum, the resultant free fatty acids contributing to the skin’s acidity, an important antimicrobial factor [55,56]. Moreover, the microbiome blocks the colonization of the skin by pathogenic microorganisms, acting as a physical barricade, as a competitor for nutrients, and a major producer of AMPs and proteases that hinder biofilm formation and impede skin colonization [57].

Apart from the complex intra- and interspecies communication, there is an intense, yet poorly understood, cross-talk between the microbiome and the host which results in a continuous modulation of the composition and virulence of the microbiome by the host and vice versa, the modification of the host’s gene expression by the microbiome-released molecules [58]. This symbiotic relationship is reciprocally advantageous and ensures the stability of the microbiome [57,59]. Disruption of this balance may lead to infections, inflammation, and autoimmunity [57].

Another major function of the microbiome is the stimulation and modulation of innate immune responses against pathogens by promoting the secretion of cytokines, especially IL-1α, receptors for complement components (C5a receptor), and AMPs, such as LL-37, β-defensin, and perforin-2 upon binding of microbiome-secreted molecules to Toll-like receptors (TLRs) and other PRRs [57,60].

The microbiome also influences the adaptive immune responses. Whereas the presence of the commensal flora does not trigger immune reactions as it hardly exerts any cytotoxicity, it serves as a permanent stimulating factor for T regulatory cells (Tregs) responses [61]. The skin microbiome promotes the cutaneous accumulation and activity of CD4+ Tregs, although to a lesser extent compared with the gut microbiome [62,63]. This ensures immune tolerance to commensal microorganisms [63].

*Corynebacterium accolens* induces the proliferation of cutaneous IL-17-producing γδ T cells [64], protective against *Staphylococcus epidermidis* and *Candida albicans*. This highlights the role of the skin microbiome in psoriasis onset or exacerbation as it has been demonstrated that γδ T cells are capable of secreting IL-17A in the absence of IL-23 stimulation [65]. Commensal bacteria can also activate IL-17A-producing cytotoxic (TC17) and helper (Th17) T cells that become tissue-resident memory cells and participate in the defense against pathogens and tissue repair [66].

Recent studies have shed light on the bidirectional relation between the skin microbiome and the microbiome of other organs, particularly the intestinal microbiome, leading to the concept of the gut–skin axis [67]. Skin exposure to environmental factors was proven to influence the intestinal microbiome. Photoexposure increases the diversity of the gut microbiome and promotes proliferation of *Lachnospiraceae* spp., *Lachnopsira* spp., and *Fusicatenibacter* spp., at least partly through the increase in serum 25-hydroxyvitamin D levels [68]. Exposure of the skin to household dust increases the likelihood of food allergies given the immunoglobulin (Ig) E-induced mast cell proliferation in the digestive tract [69]. Cutaneous chronic wounds are characterized by hyaluronan catabolism, which perturbs the function of intestinal fibroblasts and alters the gut microbiome, leading to intestinal inflammation [70]. On the other hand, the intestinal microbiome protects the skin homeostasis by acting as a barrier for invading bacteria that could otherwise enter the bloodstream by releasing anti-inflammatory and immune-modulating metabolites, such as retinoic acid, polysaccharide A, and short-chain fatty acids (SCFAs) [57,71].

Considering the delicate equilibrium between the host and the skin microbiome and the importance of the latter in maintaining skin homeostasis, it is not surprising that even subtle disturbances of the microbiome may trigger the onset or exacerbation of local or systemic inflammatory and autoimmune diseases, such as psoriasis, atopic dermatitis, acne vulgaris, hidradenitis suppurativa, seborrheic dermatitis, and alopecia areata [19]. The underlying mechanisms are only starting to be unveiled. They include loss of immune tolerance to commensal skin flora, disrupted balance between Tregs and effector cells, and excessive Th17 polarization [19,24].

## 3. The Changes in the Skin Microbiome in Psoriasis and Its Role in Psoriasis Pathogenesis

Infectious agents have been long acknowledged as potential triggers for psoriasis in genetically predisposed individuals. In children and young adults, the main eliciting factors for guttate psoriasis are pharyngeal infections with group A beta-hemolytic streptococci or perianal streptococcal infections [72]. Due to molecular mimicry between the M protein present on the surface of *Streptococcus pyogenes* and type I keratin, autoreactive T cells are activated and an intense Th1 immune response ensues [72]. In addition, a series of other bacteria (*Staphylococcus aureus*), viruses (HPV and endogenous retroviruses), and fungi (*Malassezia* spp. and *Candida albicans*) have been shown to trigger psoriasis through Th17 polarization and proinflammatory effects and to influence its course [73,74].

Complexes formed by LL-37 cathelicidins and DNA of apoptotic epithelial cells are recognized by TLR-9 on plasmacytoid dendritic cells (pDCs), which secrete large quantities of IFN-α, leading to the activation and maturation of myeloid dendritic cells (mDCs) [75]. Additionally, LL-37–RNA complexes also activate pDCs through TLR-7 and mDCs through TLR-8, prompting the release of TNF-α, IL-12, IL-23, and inducible nitric oxide synthase (iNOS) [75]. Activated mDCs migrate to the regional lymph nodes and induce the differentiation of naive T cells into Th1 and Th 17 cells, the major mediators of psoriasis pathogenesis [76]. In support of the importance of skin commensal bacteria in the pathogenesis of psoriasis are the results of the study conducted by Kolbinger et al., who showed that serum and cutaneous β defensin levels correlate with those of IL-17 and disease severity and decrease following treatment with anti-IL-17 monoclonal antibodies [77].

**Figure 3:**
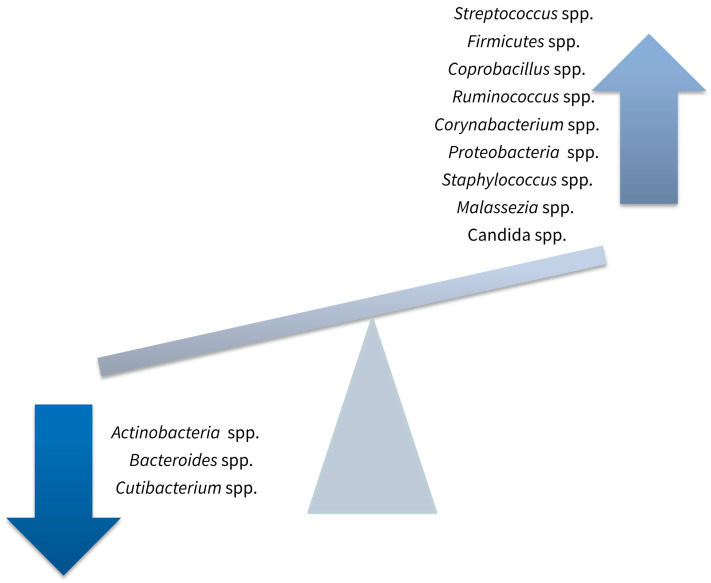
Changes in skin microbiome in psoriasis.

Studies carried out so far yielded contradicting results regarding the changes in the skin microbiome in psoriasis patients due to different technologies employed to assess the microbiome composition and lack of control for cofounder factors. Results may also vary depending on the sampling techniques used in determining the qualitative and quantitative changes in the cutaneous microbiome. Culture-based sampling methods present lower detection rates than culture-independent sampling methods. The cutaneous tissue is influenced by external factors such as ambient temperature, urban or rural environments, geographical localization, or hygienic habits prone to multiple variables. Depending on the external factors or interindividual variations, results may be contradictory [78,79]. Another important variable that could lead to contradictory results is represented by the method used in collecting the skin samples. Important differences were observed between swab sampling and cutaneous punch biopsies concerning the richness of the skin microbiome in favor of the swab sampling method [79]. Depending on the depth of the sampling method, the assessment of the skin microbiome may vary [34]. Adhesive tape sampling and skin biopsies could approach cutaneous microorganisms such as bacteria or fungi from deeper layers of the skin, including pores and sebaceous glands [80]. However, a few conclusions may be drawn. The concentrations and distribution of commensal microorganisms in psoriasis lesions and nonlesional skin of psoriasis patients show considerable differences compared to healthy subjects [80,81,82]. Psoriasis is associated with a more heterogeneous and unstable skin microbiome [80,81,82]. *Streptococcus* spp. and *Firmicutes* spp. are particularly prevalent on the skin of psoriasis patients [80,81,82,83,84]. While *Coprobacillus* spp., *Ruminococcus* spp. [83,84], *Corynebacterium* spp. [81,85,86,87] and *Proteobacteria* spp. [82,88] have been isolated in higher concentrations from the lesional and nonlesional skin of psoriasis patients than healthy individuals, *Actinobacteria* spp., *Bacteroides* spp., and *Cutibacterium* spp. are encountered less frequently (Figure 3) [82,83,84,85]. Colonization of psoriasis lesions and nonlesional skin of psoriasis patients with *Staphylococcus* spp. has also been reported by several research teams [80,81,89,90,91], but contradicted by others [82].

The increased diversity of the skin microbiome observed in psoriasis patients is not limited to bacteria, but also involves fungi [92]. Controversy persists regarding the changes in *Malassezia* spp. density on the skin of psoriasis patients. Some authors reported reduced counts of *Malassezia* spp. in psoriasis lesions [92], while others detected increased numbers of *Malassezia* spp. during disease exacerbations, particularly *Malassezia restricta* and *Malassezia globosa* [93,94]. *Malassezia* spp. may play a role in the pathogenesis of psoriasis given its effect on keratinocytes. It promotes the release of transforming growth factor (TGF)-β1, integrins, and heat shock protein (HSP) 70, thus stimulating immune cell migration and sustaining the proliferation of keratinocytes [95]. Moreover, *Malassezia* spp. produces neutrophil chemoattractants that are sometimes present in large numbers in psoriasis lesions, creating Munro’s microabscesses [25,76]. *Candida albicans* has also been isolated from psoriasis lesions, being abundant in inverse psoriasis lesions (Figure 3) [96]. Candida-sensitized αβ T cells produce IL-17, contributing to the persistence of the disease and to psoriasis flares [76,97,98,99]. *Candida* spp. may also be involved in the development and persistence of pustular psoriasis as it secretes β-glucan, which is subsequently recognized by PRRs on mDCs and stimulates the production of IL-36α [100]. As previously mentioned, IL-36 produced during the innate immune response to commensal flora is an important player in the pathogenesis of pustular psoriasis [101].

Some viruses have also been studied as potential exacerbating factors for psoriasis. Infection with human immunodeficiency virus (HIV) and HPV are associated with more severe forms of psoriasis, probably by stimulating the release of substance P, a well-known inducer of keratinocyte proliferation [24,76].

## 4. The Influence of Psoriasis Treatments on the Skin Microbiome

In addition, psoriasis treatments modulate the skin microbiome. Apart from its immunosuppressive effects, narrow-band ultraviolet B therapy (nb-UVB) beneficially influences skin microbiome by improving the oxidative stress [102,103]. The DNA damage caused by ultraviolet radiation activates a series of intracellular signaling pathways that stimulate melanogenesis. The antioxidant properties of melanin are well known [104]. Certain bacteria, such as *Streptomyces glaucescens* [105] and fungi, such as *Malassezia* spp., *Cladosporium* spp. [106], *Sporothrix Schenckii* [107], and *Cryptococcus neoformans* [108], also produce melanin as a mechanism of protection from ultraviolet radiation, further reducing oxidative stress. Nb-UVB stimulates the synthesis of vitamin D, which exerts modulatory effects on the gut and skin microbiome through incompletely elucidated mechanisms [68,109]. Several recent studies have demonstrated that topical calcipotriol triggers the release of cathelicidin, an AMP that inhibits *Malassezia* growth [110,111].

Balneotherapy impacts the composition of skin microbiome. Martin et al. showed that it increases the number of *Xanthomonadaceae* spp. of the genus *Proteobacteria* [112]. Manara et al. also studied the effects of balneotherapy on the skin microbiome of psoriasis lesions and observed a marked tendency to restoration of the normal-skin microbiome. Thermal treatment lead to a considerable reduction in bacteria previously isolated from psoriasis lesions but not from normal skin, such as *Ornithinimicrobium*, *Mesorhizobium*, and *Thermus*, as well as an increase in bacteria that were found in low numbers in psoriasis lesions before treatment, such as Delftia, Gordonia, and *Cloacibacterium*. These changes were associated with clinical improvement supporting the hypothesis that psoriasis severity depends, among other factors, on the skin microbiome composition [113].

Antibiotics required for the management of superinfected psoriasis lesions lead to clinical improvement [114,115], but their use to correct dysbiosis is not justified given their undesirable effects on normal cutaneous and intestinal flora [85].

The effect of biologic therapies used for the treatment of psoriasis on the skin microbiome has been investigated in a small number of studies. These therapies influence the composition of the skin microbiome, especially the *Actinobacteria* spp./*Firmicutes* spp. ratio [116]. While anti-TNF α agents are associated with the highest risk of severe cutaneous infections [117], mucosal candidiasis is most commonly encountered in patients receiving anti-IL-17 therapies [118]. The anti-IL-17 monoclonal antibody secukinumab also seems to have the most pronounced effect on cutaneous commensal bacteria, increasing the concentration of *Proteobacteria* spp., *Enterobacteriacea* spp., and *Pseudomonadaceae* spp. and decreasing that of *Firmicutes* spp. and *Bacteroidetes* spp. [119]. In a study conducted by Aksoy et al., the results outlined that the population of *Demodex* spp. increases in patients treated with biological therapy [115] Moreover, the intestinal microbiome in psoriasis patients who respond to secukinumab considerably differs from that of nonresponders, indicating gut microbiome as a potential biomarker for secukinumab efficacy in psoriasis [119].

Treatment with ustekinumab, an anti-IL-12/23 monoclonal antibody, is associated with a significant reduction in fungal diversity and a decrease in *Malassezia* spp. counts, as well as an increase in the numbers of *Agrobacterium* spp., *Caulobacteraceae* spp., and *Pseudomonas* spp. and a decrease in *Staphylococcus epidermidis* in moist skin regions like the antecubital fossa and axilla, but does not influence the microbiome in sebum-rich areas or mucosal surfaces [120,121]. It has been hypothesized that anti-IL-12/23 and anti-IL-23 antibodies inhibit the release of AMPs, allowing microbial variance [122].

## 5. Modulation of the Skin Microbiome as an Adjuvant Treatment in Psoriasis Patients

Considering their immunomodulatory and anti-inflammatory potential and their beneficial effect on skin barrier integrity demonstrated by numerous studies [114,123], the benefit of administering pro- and prebiotics in psoriasis patients and their ability to maintain the homeostasis of the skin microbiome has been investigated [124,125]. Although evidence stems from very few studies, the results are promising [126,127,128]. In the study conducted by Navarro–López et al., the administration of mixed probiotics (*Bifidobacterium longum*, *Bifidobacterium lactis*, and *Lactobacillus rhammosus*) was associated with enhanced response to treatment, clinical improvement of psoriasis lesions, and fewer recurrences [129]. Groeger et al. studied the effect of the oral administration of *Bifidobacterium infantis* for 6–8 weeks in psoriasis patients and reported a considerable decrease in the levels of inflammatory biomarkers, TNF-α and IL-6 [130]. Likewise, supplementation of psoriasis patients’ diet with *Lactobacillus* strains for 8 weeks decreased the levels of inflammatory biomarkers, IL-6, and malondialdehyde and increased the total antioxidant capacity [131]. A case of severe refractory pustular psoriasis significantly improved after only 2 weeks of *Lactobacillus sporogenes* orally administered thrice daily [132]. A reduction in cytokine levels (TNF-α, IL-6, IL-23, IL-17, and IL-22) and improvement in psoriasis lesions was also achieved in animal studies with oral administration of *Lactobacillus pentosus* GMNL-77 and *Lactobacillus sakei proBio-65* [133,134]. Psoriasis patients also benefit from the administration of *Prevotela histicola* formulation EDP1815, as proven by the results of a phase 2 clinical trial [135]. In the study of Ahmed et al., carried out on psoriasis patients with *Helicobacter pylori* infection, treatment administrated for *Helicobacter pylori* lead to decreasing values for psoriasis area severity index (PASI) [136].

Prebiotics also seem to have beneficial effects in psoriasis [24]. Buhas et al. evaluated the effect of a 12-week diet supplementation with a spore-based probiotic combined with a prebiotic mixture as an adjuvant treatment in psoriasis patients and reported improvement of psoriasis severity scores and a decrease in serum uric acid levels [137].

The utility of postbiotics in the treatment of psoriasis has also been studied. Postbiotic butyrate is a particularly interesting adjuvant treatment option as it has been shown to inhibit proinflammatory cytokines and stimulate the proliferation of Tregs and the differentiation of naïve CD4+ T cells into Tregs, thus preventing excessive inflammatory responses to the cutaneous commensal flora [138]. The number and function of Tregs are altered in psoriasis. Schwarz et al. and Krejner et al. demonstrated that topical sodium butyrate normalizes Tregs’ suppressive function, lowers the expression of IL-17 and IL-6, and increases the expression of IL-10 and IL-18 [139,140].

Taking into account the constant mutual influence between the gut and skin microbiomes, the frequent association of psoriasis with inflammatory bowel disease, and the impact of gut dysbiosis on the course of psoriasis, the use of fecal microbiota transplantation (FMT) in psoriasis patients has been contemplated. Yin et al. successfully applied FMT in a patient suffering from both psoriasis and irritable bowel syndrome [141].

## 6. Conclusions

Mounting compelling evidence indicate that skin dysbiosis plays a significant role in the development and exacerbation of psoriasis. The mechanisms underlying this association include the loss of immune tolerance to commensal skin flora that leads to inflammation and aggravates oxidative stress, the altered balance between Tregs and effector cells, and the excessive Th1 and Th17 polarization. The progress in the characterization of the skin microbiome in psoriasis patients has opened a whole new avenue of research focusing on the modulation of the skin microbiome as an adjuvant treatment for psoriasis and as part of a long-term plan to prevent disease flares. In addition, in individuals with a significant predisposing genetic background, the changes in the skin microbiome may help predict disease onset. The skin microbiome may also represent a valuable predictive marker of treatment response and may aid in the selection of the optimal personalized treatment. Further studies are needed in order to clarify the implication of the skin microbiome in psoriasis pathogenesis and to assess the efficacy of the different skin microbiome modulation strategies as part of the therapeutic approach of psoriasis patients. Although the oral and topical use of pre-, pro-, and postbiotics has proven beneficial in psoriasis, multiple impediments need to be surpassed. Future directions of research include strategies to avoid potential complications such as hypersensitivity reactions and dyspepsia triggered by pre- or postbiotics, infections, and exaggerated immune responses caused by probiotics. The most efficient formulations and route of administration of these agents for the prevention and adjuvant treatment of psoriasis are yet to be determined. Other appealing therapeutic approaches that are currently being explored encompass skin microbiota transplantation, skin bacteriotherapy, and therapeutic textiles, all of which have the potential to correct skin dysbiosis and reduce oxidative stress and inflammation. Future research should also explore the optimal individualized therapeutic regimen combining specific psoriasis treatments and microbiome modulators.

## Data Availability

Not applicable.
